# Midwives’ engagement in smoking- and alcohol-prevention in prenatal care before and after the introduction of practice guidelines in Switzerland: comparison of survey findings from 2008 and 2018

**DOI:** 10.1186/s12884-019-2706-8

**Published:** 2020-01-13

**Authors:** Sakari Lemola, Anna Gkiouleka, Natalie Urfer-Maurer, Alexander Grob, Katharina Tritten Schwarz, Yvonne Meyer-Leu

**Affiliations:** 1 Department of Psychology, University of Warwick, UK, University Road, Coventry, CV4 7AL UK; 2 Department of Psychology, University of Basel, Missionsstrasse 62, 4055 Basel, Switzerland; 3 Department of Health Professions, Bern University of Applied Sciences, Murtenstrasse 10, CH-3008 Bern, Switzerland; 4 School of Health Sciences (HESAV), Avenue de Beaumont 21, CH-1011 Lausanne, Switzerland; 5 University of Applied Sciences and Arts, Western Switzerland (HES-SO), Av. de Provence 6, CH-1007 Lausanne, Switzerland

**Keywords:** Smoking prevention, Alcohol use prevention, Perinatal care, Midwives, Midwifery

## Abstract

**Background:**

Evidence suggests that cigarette smoking and alcohol consumption during pregnancy negatively impacts fetal health. Health agencies across countries have developed specific guidelines for health professionals in perinatal care to strengthen their role in smoking and alcohol use prevention. One such example is the “Guideline on Screening and Counselling for prevention of cigarette smoking and alcohol consumption before, during, and after pregnancy” introduced by the Swiss Midwives Association in 2011. The current study assesses the changes in midwives’ engagement in smoking and alcohol use prevention before (2008) and after the introduction of the Guideline (2018). Further, the current study examines differences across regions (German vs. French speaking regions), graduation years (before and after the introduction of the Guideline) and different work settings (hospital vs. self-employed).

**Methods:**

Survey data were collected in 2008 (*n* = 366) and in 2018 (*n* = 459). Differences in how midwives engaged in smoking and alcohol use prevention between 2008 and 2018 were assessed with chi-square tests, as were differences across German and French speaking regions, graduation years (before and after the introduction of the Guideline) and across different work settings (working in hospitals or as self-employed).

**Results:**

An increase in midwives’ awareness of the risks of consuming even small quantities of cigarettes and alcohol for the unborn child between 2008 and 2018 is evident. Explaining the risks to pregnant women who smoke or use alcohol remained the most frequently reported prevention strategy. However, engagement with more extensive smoking and alcohol use preventive strategies across the whole course of pregnancy, such as assisting women in the elaboration of a plan to stop smoking/alcohol use, remained limited.

**Conclusions:**

Seven years after its introduction, the effectiveness of the Guideline in increasing midwives’ engagement in smoking and alcohol use prevention appears limited despite midwives’ increased awareness.

## Background

There is a large body of evidence to show that cigarette smoking and alcohol consumption during pregnancy negatively impacts fetal health [1, 2]. Studies systematically report that smoking during pregnancy is associated with preterm delivery, low birth weight, and spontaneous abortion [3]. Similarly, higher levels of alcohol consumption in pregnancy is associated with negative birth outcomes and increased risk of neurodevelopmental disorders for the child [1, 4].

Since smoking and alcohol use related risks for prenatal and neonatal health are avoidable, national and international health institutions have increasingly emphasized the important role of healthcare professionals, especially midwives, in smoking and alcohol use prevention among pregnant women [5, 6]. Midwives care for women during and/or after pregnancy, and are thus well-positioned for screening the smoking and alcohol use habits of women, and observing their exposure to passive smoke. Moreover, through building trusting and supportive relationships, midwives can effectively educate pregnant women about relevant risks for the child and can facilitate positive changes to women’s behavioral patterns [7, 8]. Empirical evidence shows that in high-income countries, psychosocial interventions increase the rates of women who quit smoking during late pregnancy [9]. Further, counselling on smoking cessation has been shown to be particularly effective when it is used consistently throughout the course of pregnancy [10]. In accordance, health agencies across countries have developed specific guidelines for health professionals in order to strengthen their role in perinatal smoking and alcohol use prevention.

In 2005, a survey-based study commissioned by the Swiss Federal Office of Public Health (FOPH) revealed that around 10% of the pregnant women in Switzerland smoked daily, while 30% drank alcohol at least once per month, and 2.2% reported consuming at least four drinks on a single occasion at least once during pregnancy [11–13]. Another study examined pregnant women’s alcohol use in 11 European countries and found that in Switzerland, alcohol consumption during pregnancy is higher (20.9% of women) than in most other studied countries, including Norway (4.1%), Sweden (7.2%), and Poland (9.7%) [14]. Furthermore, only around 66% of pregnant women reported having been screened for smoking during pregnancy and around 36% reported having received screening for alcohol consumption during prenatal care [11–13], which suggests a lack of effective smoking and alcohol use prevention services in Switzerland.

In response to these figures and in accordance with the national strategy for the prevention of non-communicable diseases in Switzerland [15], the Swiss Midwives Association introduced the “Guideline on Screening and Counselling for prevention of cigarette smoking and alcohol consumption before, during, and after pregnancy” in 2011 (revised and updated version released in 2017). Its goal was to provide guidance for midwives’ everyday practice regarding screening and consultation on cigarette and alcohol consumption among women before, during and after pregnancy [16, 17]. The practice Guideline includes a decision tree to facilitate decision-making and integrates smoking and alcohol prevention into the midwives’ work routine suggesting the following action points: (1) Universal screening of pregnant women regarding smoking and alcohol use; (2) Informing all women about the smoking and alcohol use related risks for the child; (3) Recommending smoking cessation; (4) Recommending abstinence from alcohol during pregnancy; (5) Addressing the risks related to environmental smoke exposure; (6) Integrating counselling and/or intervention approaches including motivational interviewing [18] and the ‘5A’ approach [19]; and (7) Referrals to specialist care for women with heavier smoking and/or alcohol use habits [16, 17]. Similar guidelines have been implemented in other countries including the Netherlands [20, 21], Scotland [22], Australia [23], and Canada [24]. However, recent evaluation studies provide mixed findings regarding the extent with which they are implemented consistently [21–27].

The aim of the current study was to evaluate the extent to which midwives increased their engagement in smoking and alcohol use preventive activities after the Guideline was introduced. To achieve this aim, we assessed the change in midwives’ screening and counselling practice in Switzerland, between 2008 (when the first survey with midwives was conducted, prior to the introduction of the Guideline) and 2018 (when a second survey was conducted including the same questions). Moreover, we examined changes in midwives’ awareness of smoking and alcohol consumption related risks for the child as well as their perceived barriers to effective smoking and alcohol use screening and prevention. As a further aim of the study, we examined the extent to which engagement in smoking and alcohol use prevention in 2018 was related to (a) whether midwives graduated before or after the introduction of the Guideline in 2011, (b) regional differences (i.e. between the French and German speaking regions in Switzerland), and (c) differences across work settings (i.e. working in hospitals versus being self-employed).

## Methods

### Study settings & respondents

In 2008, all midwives listed in the public phone book of Switzerland were contacted via regular mail and questionnaires were sent in their respective national languages (German, French, and Italian). In total, 1270 midwives were contacted, of whom 366 participated in the study (28.8% of all the midwives registered in the phone book) [8]. In January 2018, the Swiss Association of Midwives provided us with the e-mail addresses of its members. In total, 3136 midwives were contacted, of whom 661 completed an online questionnaire (21.1% of all the members of the Swiss Association of Midwives; it is possible that the response rate was lower in 2018 as the Members’ list of the Swiss Midwife Association may have included individuals who were no longer practicing as midwives). All participants were assured that their email addresses were only to be used for recruitment reasons for the present study and that their data was anonymized. In the paper-based survey in 2008, participants gave written informed consent, while in the online survey in 2018, informed consent was provided by clicking a respective button.

In 2008, participants answered a questionnaire regarding their screening and counselling activities during prenatal care. In 2018, participants completed mostly the same questionnaire if they were mainly involved in prenatal, or in both prenatal and postnatal care. Participants who indicated that they were exclusively involved in postnatal care in 2018 completed a questionnaire with the same content but phrased according to their screening and counselling activities with women after childbirth (*N* = 202). The study received ethical approval by the Institutional Review Board of the Department of Psychology of the University of Basel as well as the Humanities and Social Sciences Research Ethics Sub-Committee of the University of Warwick (158/17–18) and complies with the standards of the Declaration of Helsinki [28].

For comparisons between 2008 and 2018, we identified a subsample of midwives from both surveys according to the following criteria: i) being involved in prenatal care; ii) having their first consultation with the pregnant women before the 38th gestational week; and iii) had built a longer-term relationship (e.g. accompanying the women through pregnancy). Midwives who were solely involved in birth preparation services or postnatal care were excluded from these analyses. In 2008, 227 of the 366 participating midwives met the above inclusion criteria (62.0%). In 2018, 459 of the 661 midwives answered the questions regarding prenatal care of which 300 (65.4%) met the inclusion criteria. The sample characteristics are presented in Table 1. The samples at both time points were comparable and included respondents who were similar in age, years after graduation, work settings (hospital vs. midwife practice), and geographical region (German vs. French speaking Switzerland).

**Table 1:** Sample Characteristics

	Year of study: 2008 (*n* = 227)	Year of study: 2018 (*n* = 300)	Chi^2^/t-test	*p*
Age M (SD)	45.17 (7.73)	42.76 (10.12)	2.968	0.003
Female gender (%)	226 (100)	298 (99.3)	1.512	0.219
Years since graduation *M (SD)*	19.40 (9.04)	16.53 (9.80)	3.433	0.001
Employment setting^a^				
Hospital only (%)	60 (26.4)	89 (29.8)	0.655	0.418
Hospital & additional setting (%)	81 (35.8)	NA		
Self-employed only (%)	103 (45.4)	193 (64.5)		
Self-employed & additional setting (%)	145(64.2)	NA		
Doctor’s practice only (%)	2 (0.9)	4 (1.3)		
Other facility only (%)	18 (7.9)	13 (4.3)		
Hospital & Self-Employed (%)	14 (6.1)	NA		
Hospital & Doctor’s practice (%)	1 (0.4)	NA		
Self- employed & Other facility (%)	23 (10.1)	NA		
Self-employed & Hospital & Doctor’s practice (%)	2 (0.9)	NA		
Self-employed & Hospital & Other facility (%)	4 (1.8)	NA		
Number of pregnancy checks *M (SD)*	5.36 (3.90)	5.32 (5.75)	0.088	0.930
Gestational week of 1st pregnancy check *M (SD)*	17.18 (8.01)	19.11 (7.50)	−2.852	0.005

*n* of participants meeting pregnant women for the first time before the 38th gestational week

^a^ In the 2008 assessment multiple responses were possible, i.e. midwives could indicate to be employed in more than one setting. In the 2018 assessment only one response option was possible (i.e. midwives indicated only their main employment setting). Additional Chi^2^ calculations were conducted comparing ‘hospital & additional setting’ in 2008 [81 (35.8%)] with ‘hospital’ in 2018 [89 (29.8)] that revealed a non-significant difference: Chi^2^ (1) = 2.169, *p* = 0.141; and comparing ‘self-employed & additional setting’ in 2008 [145(64.2%)] with ‘self-employed’ in 2018 [193 (64.5%)] again with a non-significant difference Chi^2^ (1) = 0.009, *p* > 0.927

### Measures

The participants were asked questions that covered the seven components of the Guideline (i.e. their engagement in smoking and alcohol consumption screening and prevention) as well as additional questions about their perceptions regarding smoking and alcohol use related risks for the unborn child, and a self-evaluation of their effectiveness at smoking and alcohol use prevention. Specifically, they had to indicate whether they perceived smoking and alcohol use to be risky and to what extent (e.g. ‘*How do you assess the risks of 1-2 cigarettes consumption per day during pregnancy for the child?’* and ‘*How do you assess the risks of drinking one glass of alcohol per day during pregnancy for the child?’* answered with the options ‘*harmless’; ‘slightly increased risk for the child’; ‘significantly increased risk for the child’*); whether they routinely asked all women about their smoking and alcohol use habits (e.g. ‘*Do you routinely ask pregnant women if they smoke cigarettes?’* and ‘*Do you routinely ask pregnant women whether they consume alcohol?’* answered with the options *‘I ask every pregnant woman if she smokes/uses alcohol’*; *‘I ask when I suspect a pregnant woman of smoking/alcohol use’*; *‘I do not ask pregnant women if they smoke/use alcohol’*); whether they implemented specific interventions when women reported smoking or alcohol use (e.g. *‘How do you intervene when a pregnant woman claims to smoke/consume alcohol during pregnancy?*’ with answers e.g. *‘I explain in detail the risks of smoking/alcohol use for the child’*); and whether they asked about women’s exposure to passive smoking and their partner’s smoking and alcohol use habits (e.g. *‘Do you ask pregnant women if their partner smokes/uses alcohol?’* answered with *‘Yes, I ask if her partner smokes/uses alcohol’* or *‘No, I do not ask if her partner smokes/uses alcohol’*). Finally, they had to answer questions about potential barriers that restrained them from addressing smoking and alcohol consumption in their everyday practice.

To improve comparability between the surveys from 2008 and 2018 the same answer format was used. Questions that were answered with four-point scales were recoded into binary variables (e.g. the item ‘Perceived importance of partner’s smoking’ took 1 if the respondents had chosen ‘rather high’ or ‘very high’, and 0 if the respondents had chosen ‘rather low’ or ‘very low’) to communicate the results more effectively.

### Analysis

In order to evaluate differences in attitudes and engagement in smoking and alcohol use prevention between 2008 and 2018, we conducted Chi-square tests for categorical variables (significance <.05). Moreover, in further analyses of the 2018 sample, we conducted Chi-square tests to evaluate differences between German and French speaking regions, differences between midwives who had graduated before or after the introduction of the Guideline using the year 2012 as the cut-off point, and between midwives working in different settings (i.e. working in hospitals vs. self-employed midwives).

Due to minor differences in the survey design between 2008 and 2018, direct comparison of the responses was not possible for three items. First, in the 2008 survey, midwives could choose multiple responses regarding their employment setting (i.e. midwives could indicate that they were employed in more than one setting), while in the 2018 survey, only one response option was possible (i.e. midwives indicated only their main employment setting). Hence, further Chi-square tests compared ‘working in a hospital & additional setting’ in 2008 with ‘working in a hospital’ in 2018; and ‘self-employed & additional setting’ in 2008 with ‘self-employed’ in 2018. Second, in the 2018 survey, multiple responses were possible for smoking-related advice items (i.e. midwives could indicate both ‘advising to quit smoking’ and ‘advising to reduce smoking’ as answers). Due to non-comparability of the response options, no statistical comparisons were conducted for that question. Finally, in the 2018 survey, multiple responses were possible regarding alcohol consumption advice (i.e. midwives could indicate that they advised both ‘strict abstinence’ and ‘up to sipping from the glass’), but only one response option was provided in 2008. Hence, the Chi-square calculation compared respondents who reported ‘strict abstinence’ as sole advice given regarding alcohol consumption in 2008 and 2018.

## Results

### Smoking related screening and Counselling

Table 2 shows the differences in midwives’ engagement in smoking prevention in prenatal care between 2008 and 2018 alongside the Chi-square test results. In 2018, midwives were more aware of the risks of smoking, particularly regarding smoking less than 10 cigarettes a day. For example, in 2018, 31.8% of the participating midwives considered smoking 1–2 cigarettes per day as highly risky compared to only 10.3% in 2008. Moreover in 2018, 85.6% of midwives indicated that smoking 3–9 cigarettes per day was highly risky compared to 71.4% in 2008. The extent to which passive smoking was considered risky for the child remained large, whereby approximately 95% of the respondents in each study year reported that it involves a risk. ‘Screening all pregnant women regarding their smoking habits during pregnancy’ was consistently reported by the majority of midwives, with 89.4 and 89.3% routinely engaging with this practice in 2008 and 2018 respectively. Similarly, the rates of screening for exposure to passive smoking and partner’s smoking remained rather stable.

**Table 2:** Smoking prevention in prenatal care in 2008 and 2018

Variables	N	Year 2008 (*N* = 227)^a^	Year 2018 (*N* = 300)^a^	Chi^2^	df	*p*
		n (%)	n (%)			
Risk perception: 1–2 cigarettes/day						
harmless for the child	519	48(21.5)	20(6.8)	47.991	2	< 0.001
slightly risky for the child		152(68.2)	182(61.5)			
highly risky for the child		23(10.3)	94(31.8)			
Risk perception: 3–9 cigarettes /day						
harmless for the child	518	0(0.0)	0(0.0)	15.695	1	< 0.001
slightly risky for the child		63(28.6)	43(14.4)			
highly risky for the child		157(71.4)	255(85.6)			
Risk perception: 10 or more cigarettes /day						
harmless for the child	520	0(0.0)	0(0.0)	N/C		
slightly risky for the child		0(0.0)	0(0.0)			
highly risky for the child		222(100)	298(100)			
Risk perception: sudden cessation						
not risky for the child	509	97(44.1)	102(35.3)	5.278	3	0.153
slightly risky for the child	509	105(47.7)	157(54.3)			
highly risky for the child	509	5(2.3)	13(4.5)			
I don’t know	509	13(5.9)	17(5.9)			
Risk perception: Passive smoking						
Environmental smoke is a risk	521	215(95.1)	285(96.6)	0.722	1	0.395
‘it is rather harmless’ & ‘I don’t know’		11(4.9)	10(3.4)			
Screening: Routinely asking all women whether they smoke						
all women	524	202(89.4)	266(89.3)	0.668	2	0.716
only those suspected for smoking	524	22(9.7)	27(9.1)			
none	524	2(0.9)	5(1.7)			
Screening: Asking about exposure to passive smoking	515	123(54.4)	134(46.4)	3.294	1	0.070
Screening: Asking whether the partner smokes	515	152(67.3)	178(61.6)	1.768	1	0.184
Perceived importance of partner’s smoking (rather or very important)^b^	520	209(92.9)	274(92.9)	0.000	1	0.997
Routinely explaining to all women the risks of smoking for the child	519	145(64.4)	155(52.7)	7.182	1	0.007
Stop smoking interventions with smokers						
Explaining the risks for the child	526	187(82.7)	257(85.7)	0.837	1	0.360
Repeatedly addressing smoking in consequent appointments	526	126(55.8)	150(50.0)	1.71	1	0.191
Assisting in elaboration of a plan to stop smoking	526	79(35.0)	114(38.0)	0.514	1	0.473
Providing information material to smokers	526	36(15.9)	69(23.0)	4.033	1	0.045
Referral to an expert	526	21(9.3)	64(21.3)	13.794	1	< 0.001
Referral to behavioral therapy	526	9(4.0)	16(5.3)	0.52	1	0.471
Agreement to quit	526	15(6.6)	11(3.7)	2.421	1	0.120
Nicotine replacement therapy	526	12(5.3)	22(7.3)	0.873	1	0.350
no intervention	526	18(8.0)	6(2.0)	10.531	1	0.001
Barriers: Reasons not to address smoking (rather or very true)^c^						
Shortage of time	445	20(9.6)	34(14.3)	2.325	1	0.127
I already know many of the women and their smoking habits from previous pregnancies	475	70(33.7)	87(32.8)	0.036	1	0.850
Most women already know the risks	486	98(46.7)	116(42.0)	1.041	1	0.308
Women with children are generally well informed about the risks	485	89(42.6)	107(38.8)	0.719	1	0.396
It is not within my area of responsibility	485	8(3.8)	12(4.3)	0.071	1	0.790
Uncertainty about clinical relevance of smoking	475	26(12.3)	36(13.6)	0.178	1	0.673
Uncertainty about being able to intervene effectively	483	68(32.4)	89(32.6)	0.003	1	0.959
Giving advice to smokers is not effective	481	107(51.2)	124(46.0)	1.300	1	0.254
Pregnant women probably do not honestly report on smoking	485	96(45.7)	125(45.5)	0.003	1	0.955
In vocational training I was not informed on the risks of smoking	490	44(20.8)	60(21.6)	0.049	1	0.824
Smoking in pregnancy is a matter of private life and should not be interfered with	490	16(7.6)	9(3.2)	4.711	1	0.030
Advice given regarding smoking^d^:						
to quit	523	109(48.9)	89(29.7)			
“to quit” & “to reduce”		NA	144(48.6)			
to reduce	523	90(40.4)	61(20.3)			
not to change	523	0(0.0)	2(0.7)			

^a^The numbers of participants in analyses differ slightly due to missing values

^b^ The answers ranged from very important to very irrelevant on a 4-point scale, we merged them into two categories: ‘rather or very important’ that took 1 and ‘rather or very unimportant’ that took 0

^c^ The answers ranged from very true to very untrue on a 4-point scale, we merged them into two categories: ‘rather or very true’ that took 1 and ‘rather or very untrue’ that took 0

^d^ In the 2018 assessment, multiple responses were possible, i.e. midwives could indicate both ‘to quit’ and ‘to reduce’ as answers. Due to non-comparability of the response options, no statistical comparisons were conducted

When asked about their actual engagement in smoking prevention, the rate of midwives who routinely explained the smoking related risks for the child to all women regardless of their smoking habits decreased from 64.4% in 2008 to 52.7% in 2018. In contrast, the number of midwives who referred smoking pregnant women to an expert increased from 9.3% in 2008 to 21.3% in 2018. Further, midwives who reported no engagement in any intervention decreased from 8.0 to 2.0%. As for more extensive interventions, no statistically significant changes were observed between the two study waves regarding addressing smoking repeatedly in subsequent consultations after the first one. Similar rates for 2008 and 2018 were also found for assisting women in the elaboration of a quit plan, provision of information material, referrals of smokers to behavioral therapy, attempts to set a smoking cessation agreement, and recommending nicotine replacement therapy.

Finally, the perceived barriers to addressing smoking during pregnancy also remained similar between 2008 and 2018, whereby the most often reported reasons were uncertainty about being able to intervene effectively, that pregnant women would not report smoking habits accurately, and that women were already well aware of the smoking related risks.

### Alcohol use related screening and counselling

Regarding prevention of alcohol use, the Chi^2^ test results (Table 3) show that midwives’ awareness of alcohol use-related risks for the child increased substantially in 2018 and particularly regarding smaller quantities of alcohol. The percentage of midwives who considered rarely sipping a glass of alcohol to be harmless decreased from 83.8% in 2008 to 60.6% in 2018. Further, in 2018, 66.4% of the midwives perceived drinking up to three glasses of alcohol per week as highly risky, and 94.7% perceived drinking one glass of alcohol per day as highly risky. In 2008, the respective rates were 24.3 and 66.5%. However, screening for alcohol use during pregnancy remained equally frequent between 2008 and 2018 and so did asking distinct questions regarding frequency, average amount and type of alcohol consumption, as well as questions regarding partners’ alcohol use habits. In contrast, the percentage of midwives asking pregnant women about binge drinking (defined as drinking 4 glasses of alcohol/occasion) increased from 8.0% in 2008 to 17.0% in 2018.

**Table 3:** Prevention of alcohol consumption in prenatal care in 2008 and 2018

Variables	N	Year 2008 (*N* = 227)^a^	Year 2018 (*N* = 300)^a^	Chi-2	df	*p*
		n (%)	n (%)			
Risk perception: rarely sipping on a glass of alcohol						
harmless	501	186(83.8)	169(60.6)	34.797	2	< 0.001
slightly risky		34(15.3)	90(32.3)			
highly risky		2(0.9)	20(7.2)			
Risk perception: 3 glasses/week						
harmless	501	50(22.9)	5(1.8)	108.889	2	< 0.001
slightly risky		115(52.8)	90(31.8)			
highly risky		53(24.3)	188(66.4)			
Risk perception: 1 glass/day						
harmless	504	4(1.8)	0(0.0)	68.274	2	< 0.001
slightly risky		70(31.7)	15(5.3)			
highly risky		147(66.5)	268(94.7)			
Risk perception: Sporadically alcohol use large amounts (4 glasses/occasion)						
harmless	503	1(0.5)	0(0.0)	2.358	2	0.308
slightly risky		8(3.6)	6(2.1)			
highly risky		211(95.9)	277(97.9)			
Screening: Routinely asking all women whether they consume alcohol						
all	508	188(83.2)	231(81.9)	0.144	2	0.931
only those suspected for alcohol use		30(13.3)	40(14.2)			
none		8(3.5)	11(3.9)			
Screening: Specific questions asked regarding alcohol						
Frequency of alcohol use occasions	497	205(90.7)	254(93.7)	1.591	1	0.207
Average amount of alcohol consumed	497	172(76.1)	203(74.9)	0.096	1	0.757
Frequency of binge drinking (4 glasses on a single occasion)	497	20(8.8)	46(17.0)	7.063	1	0.008
Type of alcoholic beverages consumed	497	92(40.7)	125(46.1)	1.47	1	0.225
Screening: Asking whether the partner uses alcohol	505	70(31.1)	78(27.9)	0.638	1	0.425
Perceived importance of partner’s alcohol use (rather or very important)^b^	500	170(76.2)	209(75.5)	0.041	1	0.839
Routinely explaining to all women the risks of alcohol consumption for the child	506	135(59.7)	176(62.9)	0.515	1	0.473
Stop alcohol drinking interventions when a woman uses alcohol:						
Explaining the risks for the child	508	181(80.1)	253(89.7)	9.344	1	0.002
Repeatedly addressing alcohol use in consequent appointments	508	98(43.4)	147(52.1)	3.86	1	0.049
Assisting in elaboration of a plan to stop or reduce alcohol use	508	31(13.7)	64(22.7)	6.652	1	0.010
Providing information material to alcohol users	508	20(8.8)	65(23.0)	18.157	1	< 0.001
Referral to an expert	508	100(44.2)	135(47.9)	0.663	1	0.416
no intervention	508	13(5.8)	15(5.3)	0.045	1	0.832
Barriers: Reasons not to address alcohol use (rather or very true)^c^:						
Shortage of time	429	19(9.4)	26(11.5)	0.524	1	0.469
I already know many of the women and their habits from previous pregnancies	460	56(27.1)	55(21.7)	1.756	1	0.185
Most women already know the risks	466	79(37.6)	89(34.8)	0.407	1	0.523
Women with children are generally well informed about the risks	462	84(40.2)	88(34.8)	1.433	1	0.231
It is not within my area of responsibility	466	10(4.8)	10(3.9)	0.224	1	0.636
Uncertainty about clinical relevance of alcohol use	459	39(18.8)	31(12.4)	3.604	1	0.058
Uncertainty about being able to intervene effectively	464	59(28.1)	70(27.6)	0.016	1	0.898
Giving advice to alcohol users is not effective	452	96(46.6)	66(26.8)	19.062	1	< 0.001
Pregnant women probably do not honestly report on alcohol use	465	117(55.7)	108(42.4)	8.232	1	0.004
In vocational training I was not informed on the risks of alcohol use	466	37(17.6)	39(15.2)	0.481	1	0.488
Alcohol use in pregnancy is a matter of private life and should not be interfered with	464	11(5.2)	6(2.4)	2.694	1	0.101
Advice given regarding alcohol consumption^d^:						
strict abstinence	509	32(14.3)	120(40.0)	42.246	1	< 0.001
“strict abstinence” & “never drink more than just sipping”		NA	45(15.7)			
“strict abstinence” & “reasonable consumption”		NA	3(1.0)			
“strict abstinence” & “never more than just sipping” & “reasonable consumption”		NA	3(1.0)			
“never drink more than just sipping”	509	75(33.6)	77(25.7)			
“never drink more than just sipping” & “reasonable consumption”		NA	14 (4.9)			
reasonable consumption (“a glass every now and then”)	509	109(48.9)	21(7.0)			
one glass/day	509	2(0.9)	0(0.0)			

^a^The numbers of participants in analyses differ slightly due to missing values

^b^ The answers ranged from very important to very irrelevant on a 4-point scale, we merged them into two categories: ‘rather or very important’ that took 1 and ‘rather or very unimportant’ that took 0

^c^ The answers ranged from very true to very untrue on a 4-point scale, we merged them into two categories: ‘rather or very true’ that took 1 and ‘rather or very untrue’ that took 0

^d^In the 2018 assessment, multiple responses were possible, i.e. midwives could indicate advising both ‘strict abstinence’ and ‘up to sipping from the glass’ (which may depend on circumstances. The Chi^2^ calculated is between respondents that have reported ‘strict abstinence’ as the only type of advice given regarding alcohol consumption in 2008 and 2018

Midwives’ engagement with alcohol use prevention for pregnant women who reported alcohol use during pregnancy increased significantly from 2008 to 2018 as identified by an increase in the rates of explaining the risk for the child, assisting in the elaboration of a quit or reduction plan, and providing information material. Further, regarding the perceived barriers to addressing alcohol use during pregnancy, midwives’ beliefs that giving advice to pregnant women is not effective decreased over time (from 46.6% in 2008 to 26.8% in 2018) as did beliefs that pregnant women do not report alcohol use habits accurately (from 55.7 to 42.4%). Finally, advising strict abstinence from alcohol use became more frequent in 2018 with 40.0% exclusively choosing this response option compared to 14.3% in 2008 (*Chi*^*2*^ (1) = 42.246, *p* < 0.001).

### Differences between midwives from different regions, with different graduation years, and of different work settings

#### Smoking related screening and counselling

Analyses of regional differences showed no significant differences between French and German speaking regions in terms of smoking screening, familiarity with the Guideline and most types of advice provided. However, in German speaking regions, midwives explained smoking-related risks for the child to smoking pregnant women more often than their counterparts in French speaking regions (89.3% compared to 70.7%), and they addressed smoking in consequent appointments more systematically (56.2% compared to 24.1% in French speaking regions). However, the patterns were reversed regarding referrals of smoking women to an expert, referrals for behavioral or nicotine replacement therapy, assisting in the development of a cessation plan as well as the use of the Stages of Change Model of behavior change (Additional file 1: Table S1).

Looking at differences between midwives who graduated before the introduction of the Guideline (i.e. till 2011) and thereafter (Additional file 1: Table S3), we observed that overall, awareness of risks, familiarity with the Guideline and engagement in smoking prevention remained similar. However, midwives who graduated before 2012 routinely explained the smoking related risks for the child to all women and stressed the issue in consequent appointments more often than midwives who graduated in 2012 onwards. However, the latter appear to use the Stages of Change Model of behavior change more systematically (12.5% compared to 1.8%). There are also some significant differences regarding the reasons reported for not addressing smoking. For midwives who graduated before 2012, the most often reported reason is the assumption that women already know the relevant risks, while for those who graduated in 2012 or later, the most often reported reason is their uncertainty surrounding their own effectiveness.

Finally, regarding differences across work settings (Additional file 1: Table S5), self-employed midwives compared to those working in hospitals seemed to feel more confident about the effectiveness of their own advice, they explained the smoking related risks for the child, they assisted smoking pregnant women in setting a cessation plan, and they screened the partner’s smoking habits more often. Moreover, they were less affected by shortage of time regarding addressing smoking and they were less concerned about pregnant women’s accuracy in answering smoking related questions.

#### Alcohol use related screening and counselling

Regarding differences across regions, we observed that midwives in French speaking regions routinely explained the alcohol use related risks for the child to all women more systematically than those working in German speaking regions (88% compared to 57.4%, Additional file 1: Table S2) and they assisted more often in setting an alcohol quitting plan. However, midwives in German speaking regions repeatedly addressed alcohol consumption in consequent appointments during pregnancy more than their counterparts in French speaking regions (57% compared to 30.8%). Finally, midwives in French speaking regions had greater knowledge regarding alcohol related fetal disorders and they advised strict abstinence from alcohol more systematically.

Regarding differences related to midwives across graduation years, there were no significant differences apart from the finding that midwives who graduated before 2012 explained the risks for the child to all women more often (Additional file 1: Table S4) and screened the average amount of alcohol consumed less often compared to midwives who graduated in 2012 or later (72.1% compared to 87.8%). Overall, midwives who worked in hospitals and those who were self-employed reported similar patterns regarding alcohol- related screening and counselling (Additional file 1: Table S6).

## Discussion

The present study aimed to evaluate the extent to which Swiss midwives increased their engagement in smoking and alcohol use prevention 7 years after the introduction of the Swiss Midwives Association’s Guideline on screening and counselling for prevention of cigarette smoking and alcohol consumption. To achieve our aim, we compared midwives’ engagement in smoking and alcohol prevention in prenatal care in 2008, before the Guideline of the Swiss Midwives Association was introduced, and in 2018. Further, we conducted analyses to explore the extent to which the observed differences among midwives in 2008 and 2018 were also subject to differences across regions, graduation years and work settings. Overall, in 2018, midwives appeared more aware of the risks of consuming even small quantities of cigarettes and alcohol for the unborn child. This was particularly pronounced in relation to alcohol use. However, engagement with smoking and alcohol consumption prevention remained rather stable at low levels particularly regarding more extensive intervention efforts, across the 10 years.

Overall, explaining the risks to pregnant women who smoke/ consume alcohol remained the most frequently reported prevention strategy (> 80%), while referring women who smoke to an expert, developing a quit or reduction plan with women who smoke/ consume alcohol, or providing women with information material remained at rather low levels despite an observed increase across the two study waves. This finding suggests that a large number of midwives were still rather reluctant to engage in more extensive smoking and alcohol use prevention interventions in 2018, which may be explained by the finding that despite the introduction of the Guideline, the midwives still considered their advice against smoking and alcohol use as relatively ineffective [7, 18, 19].

To our knowledge, the contents of the Guideline have been integrated into Swiss midwives’ formal training, through particular modules concerning smoking and alcohol consumption prevention among pregnant women and new mothers. However, the extent of training in the contents of the guidelines possibly differs across midwifery schools and regions of the country. Relatedly, we expected that the engagement in alcohol use and smoking prevention would differ according to whether midwives graduated before or after the introduction of the Guideline and between different work settings. Furthermore, we assumed that whether midwives work in a hospital or as self-employed would play a role, as contextual factors such as time pressure may differ. Still, our study showed that the emerging patterns appear to be largely consistent across regions, graduation periods and work settings, although there are few significant differences in specific screening strategies and in the reasons reported for not addressing smoking and alcohol use. Thus, the findings are only partially consistent with research in the Netherlands [25] suggesting that the implementation of smoking prevention counselling as well as the relative barriers acknowledged vary across professional groups. Also, they point to the importance of further exploring the reasons that prevent midwives in Switzerland from engaging more actively in smoking and alcohol use preventive strategies despite knowing the relevant risks.

A domain where we did find differences between work settings is the opinion that pregnant women tend to misreport their smoking and alcohol use habits. This barrier to effective screening was more often mentioned by midwives working in hospitals, who also often reported that shortage of time was a barrier to addressing smoking. As a recent study [22] showed, a relationship of trust between midwives and pregnant women facilitates more authentic and accurate disclosure of substance use behaviors. Therefore, apart from integrating routine screening of smoking and alcohol use in prenatal care, it is also important that hospital work routines allow midwives sufficient time to develop a trusting relationship with pregnant women, which in turn may also improve effective substance use prevention.

Further, in terms of knowledge of the Guideline, our findings did not show any significant differences between midwives who graduated before and after the introduction of the Guideline in 2011. This indicates that the extent to which the Guideline has been integrated into midwives’ training during the 7 years since its introduction has not been sufficient to meet the desired changes in midwives’ practice. Emphasizing smoking and alcohol prevention in midwives’ basic training should be combined with further educational activities that highlight the necessity of the Guideline and its implementation, as well as with raising midwives’ awareness of the impact of their own advice [8].

Further, activities that aim to reduce midwives’ tendency to believe that pregnant women underreport their smoking and alcohol use habits, and to increase midwives’ motivation to actively engage with prevention [21, 29] might also be useful. Accordingly, our findings suggest that efforts should also focus on increasing midwives’ knowledge and skills to use the decision-making aids outlined in the Guideline. This could involve training in motivational interviewing and engagement in discussions around sensitive topics [21] (e.g. personal life information or substance abuse), as well as the integration of simple applications in midwives’ everyday practice, including digital applications or conventional tools like printed screening questionnaires, decision trees, and checklists. These would enable midwives to conduct the smoking and alcohol use screening more efficiently and to set quitting goals and behavior change interventions in a more structured way [29–31].

In sum, our findings indicate that the Guideline has not been particularly effective in increasing midwives’ engagement in smoking and alcohol consumption prevention, since the changes between 2008 and 2018 were not large. Despite being more aware of the risks related to smoking and alcohol use in pregnancy, midwives still do not engage in smoking and alcohol use prevention in a consistent manner. This is particularly alarming given the increased prevalence of smoking and alcohol use during pregnancy in Switzerland [32, 33].

The present findings should be seen in the light of certain limitations. First, there were differences regarding the study design and data collection between the two study waves. In 2008, midwives with a record in the phone book completed a hardcopy questionnaire received via regular mail, while in 2018 the members of the Swiss Midwives Association were contacted by email and asked to complete the questionnaire online. Although the background information regarding the samples look similar between the two measurement time points (e.g. similar shares of participants were working in hospitals) it remains possible that there were differences between the two samples that have not been considered.

Second, in the current naturalistic study it was not possible to study a control group unexposed to the Guideline. Therefore, it is not possible to determine whether changes were actually due to the introduction of the Guideline by the Swiss Midwives Association. Third, due to anonymity of the responses it was not possible to match answers of midwives between 2008 and 2018, although it can be assumed that the samples were partly overlapping. Fourth, the use of an even-numbered Likert scale for certain items did not allow ambiguous answers to indicate an undecided response (i.e. potentially undecided respondents were forced to decide for one side in some items). Finally, it is possible that some of the answers were influenced by memory related biases or social desirability. Future research might benefit from integrating both the midwives’ as well as the clients’ reports of midwives’ smoking and alcohol use prevention in order to account for potential response biases.

## Conclusions

In conclusion, the findings indicate that 7 years after the introduction of the Guideline by the Swiss Midwives Association in 2011, midwives appear more aware of the risks of smoking and alcohol use during pregnancy. However, systematic engagement with preventive activities across the whole course of pregnancy such as assisting women in the elaboration of a plan to stop smoking/alcohol use remained relatively infrequent. This suggests that the effect of the Guideline in changing midwives’ working practice has been limited and that future research should explore the reasons explaining the stable lack of engagement particularly in more extensive interventions. Further, our findings suggest that there should be greater emphasis on increasing midwives’ familiarity with the Guideline, increasing midwives’ self-efficacy, and improving the way in which clinical tools are available to them across different work settings, particularly in hospitals. To increase the effectiveness of counselling, midwives’ vocational training may include practical training in intervention techniques such as motivational interviewing, and hospitals may adopt longer consulting sessions to enable midwives to develop a patient-centered approach and to build a relationship of trust with their clients and potentially their partners [34]. Further, conventional (e.g. printed leaflets) and digital means (e.g. screening texts or apps) may also contribute to the more structured and systematic engagement of the midwives in screening and preventive activities before, during and after the pregnancy period.

## Supplementary information

**Additional file 1:** **Table S1.** Smoking prevention in prenatal care & knowledge of the Guideline of the Swiss Midwives Association by region. **Table S2.** Prevention of alcohol consumption in prenatal care by region. **Table S3.** Smoking prevention in prenatal care & knowledge of the Guideline of the Swiss Midwives Association by graduation year (before and after 2012). **Table S4.** Prevention of alcohol consumption in prenatal care by graduation year (before and after 2012). **Table S5.** Smoking prevention in prenatal care & knowledge of the Guideline of the Swiss Midwives Association by work setting. **Table S6.** Prevention of alcohol consumption in prenatal care by work setting.

## Data Availability

The datasets used and/or analysed during the current study are available from the corresponding author on reasonable request.

## Abbreviation

FOPH: Federal Office of Public Health
