# Supplementary material for: Midwives’ engagement in smoking- and alcohol-prevention in prenatal care before and after the introduction of practice guidelines in Switzerland: comparison of survey findings from 2008 and 2018
Source: BMC Pregnancy Childbirth. 2020 Jan 13;20:31. doi: 10.1186/s12884-019-2706-8 (PMC6958580; doi:10.1186/s12884-019-2706-8)
Supplement: Supplementary file 1 — Additional file 1: Table S1. Smoking prevention in prenatal care & knowledge of the Guideline of the Swiss Midwives Association by region. Table S2. Prevention of alcohol consumption in prenatal care by region. Table S3. Smoking prevention in prenatal care & knowledge of the Guideline of the Swiss Midwives Association by graduation year (before and after 2012). Table S4. Prevention of alcohol consumption in prenatal care by graduation year (before and after 2012). Table S5. Smoking prevention in prenatal care & knowledge of the Guideline of the Swiss Midwives Association by work setting. Table S6. Prevention of alcohol consumption in prenatal care by work setting. [file 12884_2019_2706_MOESM1_ESM.docx]

| Table S1. Smoking prevention in prenatal care & knowledge of the Guideline of the Swiss Midwives Association by region | | | | | |
| --- | --- | --- | --- | --- | --- |
|  | German  (n=242) | French  (n=58) | Chi^2^ | df | p |
| Variables | Percentage in (%) | |  |  |  |
| Risk perception: 1-2 cigarettes/day  harmless for the child | 5.4% | 12.3% | 3.517 | 2 | 0.172 |
| slightly risky for the child | 62.8% | 56.1% |  |  |  |
| significantly risky for the child | 31.8% | 31.6% |  |  |  |
| Risk perception: 3-9 cigarettes /day |  |  |  |  |  |
| slightly risky for the child | 11.3% | 27.6% | 10.096 | 1 | 0.001 |
| significantly risky for the child | 88.8% | 72.4% |  |  |  |
| Risk perception: 10 or more cigarettes /day |  |  |  |  |  |
| significantly risky for the child | 100.0 | 100.0 | N/C |  |  |
| Risk perception: sudden cessation  not risky for the child | 33.0% | 44.6% | 3.703 | 3 | 0.295 |
| slightly risky for the child | 57.1% | 42.9% |  |  |  |
| significantly risky for the child | 4.3% | 5.4% |  |  |  |
| I don't know | 5.6% | 7.1% |  |  |  |
| Risk perception: Passive smoking  Environmental smoke is a risk | 97.5% | 93.0% | 2.839 | 1 | 0.092 |
| ‘I don’t know’ | 2.5% | 7.0% |  |  |  |
| Screening: Routinely asking all women whether they smoke  all women | 88.8% | 91.4% | 1.260 | 2 | 0.533 |
| only those suspected for smoking | 9.2% | 8.6% |  |  |  |
| none | 2.1% | 0.0% |  |  |  |
| Screening: Asking about exposure to passive smoking | 43.7% | 56.9% | 3.235 | 1 | 0.072 |
| Screening: Asking whether the partner smokes | 59.5% | 70.2% | 2.212 | 1 | 0.137 |
| Perceived importance of partner’s smoking (rather or very important)^1^ | 95.4% | 82.5% | 11.614 | 1 | 0.001 |
| Routinely explaining the risks of smoking for the child to all women | 52.7% | 52.6% | 0.000 | 1 | 0.988 |
| Stop smoking interventions with smokers  explaining the risks for the child | 89.3% | 70.7% | 13.135 | 1 | 0.000 |
| repeatedly addressing smoking in consequent appointments | 56.2 % | 24.1% | 19.236 | 1 | 0.000 |
| assisting in elaboration of a plan to stop smoking | 35.1% | 50.0% | 4.395 | 1 | 0.036 |
| providing information material to smokers | 21.1% | 31.0% | 2.621 | 1 | 0.105 |
| referral to an expert | 16.9% | 39.7% | 14.382 | 1 | 0.000 |
| referral to behavioral therapy | 3.7% | 12.1% | 6.461 | 1 | 0.011 |
| agreement to quit | 3.7% | 3.4% | 0.010 | 1 | 0.922 |
| nicotine replacement therapy | 4.5% | 19.0% | 14.316 | 1 | 0.000 |
| use e-cigarettes | 5.0% | 3.4% | 0.240 | 1 | 0.624 |
| no intervention | 1.7% | 3.4% | 0.769 | 1 | 0.380 |
| Barriers: Reasons not to address smoking (rather or very true)  shortage of time | 10.8% | 26.9% | 8.576 | 1 | 0.003 |
| I already know many of the women and their smoking habits from previous pregnancies | 33.5% | 30.0% | 0.224 | 1 | 0.636 |
| most women already know the risks | 45.7% | 26.4% | 6.564 | 1 | 0.010 |
| women with children are generally well informed about the risks | 42.2% | 24.5% | 5.603 | 1 | 0.018 |
| It is not within my area of responsibility | 4.1% | 5.5% | 0.209 | 1 | 0.648 |
| uncertainty about clinical relevance of smoking | 12.6% | 18.0% | 0.997 | 1 | 0.318 |
| uncertainty about being able to intervene effectively | 29.8% | 43.6% | 3.818 | 1 | 0.051 |
| giving advice to smokers is not effective | 45.6% | 47.3% | 0.048 | 1 | 0.826 |
| pregnant women probably do not honestly report on smoking | 48.9% | 31.5% | 5.292 | 1 | 0.021 |
| In vocational training I was not informed on the risks of smoking | 22.9% | 16.4% | 1.104 | 1 | 0.293 |
| smoking in pregnancy is a matter of private life and should not be interfered with | 4.0% | 0.0% | 2.283 | 1 | 0.131 |
| screening and counseling cannot be charged | 22.6% | 11.5% | 3.134 | 1 | 0.077 |
| Advice given regarding smoking:  to quit | 78.9% | 72.4% | 1.144 | 1 | 0.285 |
| to reduce | 64.9 % | 82.8% | 6.914 | 1 | 0.009 |
| Use e-cigarettes | 1.2% | 3.4% | 1.393 | 1 | 0.238 |
| not to change | 0.8% | 0.0% | 0.483 | 1 | 0.487 |
| Knowledge of the Guideline of the Swiss Midwives Association & Use of Intervention Strategies^1^ |  |  |  |  |  |
| Familiar with the guideline (yes/no) | 32.9% | 33.3% | 0.004 | 1 | 0.952 |
| Use the intervention strategies recommended in the guideline | 20.9% | 27.9% | 0.998 | 1 | 0.318 |
| Use the 'Stages of Change' model of behavior change | 1.8% | 12.0% | 12.161 | 2 | 0.002 |
| Use the '5A Method' | 3.2% | 2.0% | 0.943 | 2 | 0.624 |
| Use the method of Motivational Interviewing | 57.4% | 31.4% | 13.206 | 2 | 0.001 |
| Frequency of consulting a specialist (one to more than five times a year) | 26.6% | 24.0% | 0.146 | 1 | 0.703 |

1 These questions refer both to smoking and drinking prevention.

| Table S2. Prevention of alcohol consumption in prenatal care by region | | | | | |
| --- | --- | --- | --- | --- | --- |
|  | German  (n=242) | French  (n=58) | Chi^2^ | df | p |
| Variables | Percentage in (%) | |  |  |  |
| Risk perception: rarely sipping on a glass of alcohol  harmless | 62.6% | 51.9% | 2.122 | 2 | 0.346 |
| slightly risky | 30.4% | 40.4% |  |  |  |
| significantly risky | 7.0% | 7.7% |  |  |  |
| Risk perception: 3 glasses/week  harmless | 1.7% | 1.9% | 0.038 | 2 | 0.981 |
| slightly risky | 32.0% | 30.8% |  |  |  |
| significantly risky | 66.2% | 67.3% |  |  |  |
| Risk perception: 1 glass/day  slightly risky | 3.5% | 13.5% | 8.453 | 1 | 0.004 |
| significantly risky | 96.5% | 86.5% |  |  |  |
| Risk perception: Sporadically drinking large amounts (4 glasses/occasion)  slightly risky | 1.7% | 3.8% | 0.915 | 1 | 0.339 |
| significantly risky | 98.3% | 96.2 |  |  |  |
| Screening: Routinely asking all women whether they consume alcohol  asking all women | 81.3% | 84.6% | 2.608 | 2 | 0.271 |
| asking only those suspected for drinking | 13.9% | 15.4% |  |  |  |
| asking nobody | 4.8% | 0.0% |  |  |  |
| Screening: Specific questions asked regarding alcohol:  Frequency of drinking occasions | 93.2% | 96.2% | 0.645 | 1 | 0.422 |
| Average amount of alcohol consumed | 74.4% | 76.9% | 0.139 | 1 | 0.709 |
| Frequency of binge drinking (4 glasses on a single occasion) | 16.0% | 21.2% | 0.798 | 1 | 0.372 |
| Type of alcoholic beverages consumed | 46.1% | 46.2% | 0.000 | 1 | 0.996 |
| Screening: Asking whether the partner drinks | 26.2% | 35.3% | 1.716 | 1 | 0.190 |
| Perceived importance of partner’s drinking (rather or very important)^1^ | 77.9% | 64.7% | 3.897 | 1 | 0.048 |
| Routinely explaining the risks of alcohol consumption for the child to all women | 57.4% | 88.0% | 16.482 | 1 | 0.000 |
| Stop drinking interventions when a woman drinks:  Explaining the risks for the child | 91.7% | 80.8% | 5.532 | 1 | 0.019 |
| Repeatedly addressing drinking in consequent appointments | 57.0% | 30.8% | 11.655 | 1 | 0.001 |
| Assisting in elaboration of a plan to stop or reduce drinking | 19.6% | 36.5% | 6.964 | 1 | 0.008 |
| Providing information material to drinkers | 21.7% | 28.8% | 1.208 | 1 | 0.272 |
| Referral to an expert | 44.8% | 61.5% | 4.772 | 1 | 0.029 |
| no intervention | 6.5% | 0.0% | 3.582 | 1 | 0.058 |
| Barriers: Reasons not to address drinking (rather or very true):  Shortage of time | 10.2% | 16.3% | 1.429 | 1 | 0.232 |
| I already know many of the women and their habits from previous pregnancies | 23.7% | 13.0% | 2.499 | 1 | 0.114 |
| Most women already know the risks | 40.2% | 10.6% | 14.777 | 1 | 0.000 |
| Women with children are generally well informed about the risks | 39.8% | 12.8% | 12.335 | 1 | 0.000 |
| It is not within my area of responsibility | 4.3% | 2.1% | 0.516 | 1 | 0.473 |
| Uncertainty about clinical relevance of alcohol use | 11.7% | 15.2% | 0.428 | 1 | 0.513 |
| Uncertainty about being able to intervene effectively | 26.9% | 30.4% | 0.233 | 1 | 0.630 |
| Giving advice to drinkers is not effective | 27.1% | 25.5% | 0.050 | 1 | 0823 |
| Pregnant women probably do not honestly report on drinking | 43.3% | 38.3% | 0.388 | 1 | 0.533 |
| In vocational training I was not informed on the risks of drinking | 16.7% | 8.5% | 2.015 | 1 | 0.156 |
| Drinking in pregnancy is a matter of private life | 2.4% | 2.1% | 0.014 | 1 | 0.907 |
| Screening and counseling cannot be charged | 19.4% | 8.7% | 2.969 | 1 | 0.085 |
| Advice given regarding alcohol consumption:  strict abstinence | 52.1% | 82.7% | 16.274 | 1 | 0.000 |
| never drink more than just sipping | 50.4% | 30.8% | 6.603 | 1 | 0.010 |
| reasonable consumption | 12.0% | 13.5% | 0.089 | 1 | 0.766 |
| no recommendation | 3.8% | 0.0% | 2.065 | 1 | 0.151 |
| Familiar with fetal disorders:  Alcohol embryopathy | 90.2% | 50.0% | 48.358 | 1 | 0.000 |
| Fetal Alcohol Syndrome (FAS) | 80.8% | 96.2% | 7.333 | 1 | 0.007 |
| Fetal Alcohol Effects (FAE) | 22.2% | 48.1% | 14.455 | 1 | 0.000 |
| Alcohol Related Nervous System Disorder | 25.2% | 34.6% | 1.911 | 1 | 0.167 |
| None | 18.8% | 1.9% | 9.144 | 1 | 0.002 |
| 1 glass of alcoholic beer per day during breastfeeding encourages milk production (rather/very true) | 25.9% | 38.0% | 2.924 | 1 | 0.087 |
| 1 glass of alcoholic beer per day during breastfeeding is harmful for the baby (rather/very true) | 82.7% | 82.7% | 0.000 | 1 | 0.993 |
| 1 glass of alcoholic beer per day during breastfeeding may be recommended (rather/very true) | 5.3% | 1.9% | 1.088 | 1 | 0.297 |

| Table S3. Smoking prevention in prenatal care & knowledge of the Guideline of the Swiss Midwives Association by graduation year (before and after 2012) | | | | | |
| --- | --- | --- | --- | --- | --- |
|  | Graduation until 2011  (n=247) | Graduation after 2011  (n=53) | Chi^2^ | df | p |
| Variables | Percentage in (%) | |  |  |  |
| Risk perception: 1-2 cigarettes/day  harmless for the child | 8.2% | 0.0% | 5.140 | 2 | 0.077 |
| slightly risky for the child | 59.7% | 69.8% |  |  |  |
| significantly risky for the child | 32.1% | 30.2% |  |  |  |
| Risk perception: 3-9 cigarettes /day |  |  |  |  |  |
| slightly risky for the child | 16.3% | 5.7% | 4.015 | 1 | 0.045 |
| significantly risky for the child | 83.7% | 94.3% |  |  |  |
| Risk perception: 10 or more cigarettes /day |  |  |  |  |  |
| significantly risky for the child | 100.0 | 100.0 | N/C |  |  |
| Risk perception: sudden cessation  not risky for the child | 37.1% | 26.9% | 3.521 | 3 | 0.318 |
| slightly risky for the child | 52.3% | 63.5% |  |  |  |
| significantly risky for the child | 5.1% | 1.9% |  |  |  |
| I don't know | 5.5% | 7.7% |  |  |  |
| Risk perception: Passive smoking  Environmental smoke is a risk | 97.5% | 92.5% | 3.410 | 1 | 0.065 |
| ‘I don’t know’ | 2.5% | 7.5% |  |  |  |
| Screening: Routinely asking all women whether they smoke  all women | 88.6% | 92.5% | 0.913 | 2 | 0.634 |
| only those suspected for smoking | 9.8% | 5.7% |  |  |  |
| none | 1.6% | 1.9% |  |  |  |
| Screening: Asking about exposure to passive smoking | 49.2% | 33.3% | 4.230 | 1 | 0.040 |
| Screening: Asking whether the partner smokes | 63.7% | 51.9% | 2.506 | 1 | 0.113 |
| Perceived importance of partner’s smoking (rather or very important) | 92.6% | 94.1% | 0.143 | 1 | 0.706 |
| Routinely explaining the risks of smoking for the child to all women | 56.2% | 36.5% | 6.637 | 1 | 0.010 |
| Stop smoking interventions with smokers  explaining the risks for the child | 84.2% | 92.5% | 2.414 | 1 | 0.120 |
| repeatedly addressing smoking in consequent appointments | 52.6 % | 37.7% | 3.873 | 1 | 0.049 |
| assisting in elaboration of a plan to stop smoking | 36.8% | 43.4% | 0.796 | 1 | 0.372 |
| providing information material to smokers | 21.1% | 32.1% | 2.994 | 1 | 0.084 |
| referral to an expert | 20.2% | 26.4% | 0.991 | 1 | 0.320 |
| referral to behavioral therapy | 4.9% | 7.5% | 0.625 | 1 | 0.429 |
| agreement to quit | 3.2% | 5.7% | 0.724 | 1 | 0.395 |
| nicotine replacement therapy | 6.5% | 11.3% | 1.506 | 1 | 0.220 |
| use e-cigarettes | 4.9% | 3.8% | 0.115 | 1 | 0.734 |
| no intervention | 2.0% | 1.9% | 0.004 | 1 | 0.948 |
| Barriers: Reasons not to address smoking (rather or very true)  shortage of time | 12.2% | 22.4% | 3.301 | 1 | 0.069 |
| I already know many of the women and their smoking habits from previous pregnancies | 37.7% | 12.0% | 12.126 | 1 | 0.000 |
| most women already know the risks | 45.7% | 26.4% | 6.564 | 1 | 0.010 |
| women with children are generally well informed about the risks | 41.5% | 26.9% | 3.787 | 1 | 0.052 |
| It is not within my area of responsibility | 4.0% | 5.8% | 0.319 | 1 | 0.572 |
| uncertainty about clinical relevance of smoking | 14.9% | 8.2% | 1.530 | 1 | 0.216 |
| uncertainty about being able to intervene effectively | 29.4% | 46.2% | 5.370 | 1 | 0.020 |
| giving advice to smokers is not effective | 45.5% | 48.1% | 0.116 | 1 | 0.733 |
| pregnant women probably do not honestly report on smoking | 47.3% | 37.3% | 1.698 | 1 | 0.193 |
| In vocational training I was not informed on the risks of smoking | 24.4% | 9.4% | 5.711 | 1 | 0.017 |
| smoking in pregnancy is a matter of private life and should not be interfered with | 4.0% | 0.0% | 2.181 | 1 | 0.140 |
| screening and counseling cannot be charged | 22.6% | 10.4% | 3.604 | 1 | 0.058 |
| Advice given regarding smoking:  to quit | 75.7% | 86.8% | 3.091 | 1 | 0.079 |
| to reduce | 68.0 % | 69.8% | 0.065 | 1 | 0.799 |
| Use e-cigarettes | 2.0% | 0.01% | 1.091 | 1 | 0.296 |
| not to change | 0.8% | 0.0% | 0.432 | 1 | 0.511 |
| not any | 1.2% | 0.0% | 0.650 | 1 | 0.420 |
| Knowledge of the Guideline of the Swiss Midwives Association & Use of Intervention Strategies^1^ |  |  |  |  |  |
| Familiar with the guideline (yes/no) | 33.3% | 31.4% | 0.073 | 1 | 0.788 |
| Use the intervention strategies recommended in the guideline | 20.1% | 32.5% | 2.967 | 1 | 0.085 |
| Use the 'Stages of Change' model of behavior change | 1.8% | 12.5% | 15.461 | 2 | 0.000 |
| Use the '5A Method' | 2.8% | 4.1% | 3.020 | 2 | 0.221 |
| Use the method of Motivational Interviewing | 50.9% | 59.6% | 1.465 | 2 | 0.481 |
| Frequency of consulting a specialist (one to more than five times a year) | 22.5% | 43.5% | 8.678 | 1 | 0.003 |

| Table S4. Prevention of alcohol consumption in prenatal care by graduation year (before and after 2012) | | | | | |
| --- | --- | --- | --- | --- | --- |
|  | Graduation until 2011  (n=247) | Graduation in after 2011  (n=53) | Chi^2^ | df | p |
|  | Perecentage in (%) | |  |  |  |
| Risk perception: rarely sipping on a glass of alcohol  harmless | 62.0% | 54.0% | 1.682 | 2 | 0.431 |
| slightly risky | 30.6% | 40.0% |  |  |  |
| significantly risky | 7.4% | 6% |  |  |  |
| Risk perception: 3 glasses/week  harmless | 1.3% | 3.9% | 2.576 | 2 | 0.276 |
| slightly risky | 33.2% | 25.5% |  |  |  |
| significantly risky | 65.5% | 70.6% |  |  |  |
| Risk perception: 1 glass/day  slightly risky | 6.0% | 2.0% | 1.382 | 1 | 0.240 |
| significantly risky | 94.0% | 98.0% |  |  |  |
| Risk perception: Sporadically drinking large amounts (4 glasses/occasion)  slightly risky | 2.6% | 0.0% | 1.315 | 1 | 0.251 |
| significantly risky | 97.4% | 100.0 |  |  |  |
| Screening: Routinely asking all women whether they consume alcohol  asking all women | 81.0% | 86.3% | 0.987 | 2 | 0.611 |
| asking only those suspected for drinking | 15.2% | 9.8% |  |  |  |
| asking nobody | 3.9% | 3.9% |  |  |  |
| Screening: Specific questions asked regarding alcohol:  Frequency of drinking occasions | 93.7% | 93.9% | 0.002 | 1 | 0.962 |
| Average amount of alcohol consumed | 72.1% | 87.8% | 5.253 | 1 | 0.022 |
| Frequency of binge drinking (4 glasses on a single occasion) | 17.1% | 16.3% | 0.018 | 1 | 0.893 |
| Type of alcoholic beverages consumed | 44.1% | 55.1% | 1.940 | 1 | 0.164 |
| Screening: Asking whether the partner drinks | 28.8% | 23.5% | 0.581 | 1 | 0.446 |
| Perceived importance of partner’s drinking (rather or very important)^1^ | 74.9% | 78.0% | 0.214 | 1 | 0.644 |
| Routinely explaining the risks of alcohol consumption for the child to all women | 66.1% | 48.0% | 5.755 | 1 | 0.016 |
| Stop drinking interventions when a woman drinks:  Explaining the risks for the child | 90.0% | 88.2% | 0.148 | 1 | 0.700 |
| Repeatedly addressing drinking in consequent appointments | 53.7% | 45.1% | 1.233 | 1 | 0.267 |
| Assisting in elaboration of a plan to stop or reduce drinking | 19.5% | 37.3% | 7.523 | 1 | 0.006 |
| Providing information material to drinkers | 21.6% | 29.4% | 1.421 | 1 | 0.233 |
| Referral to an expert | 47.6% | 49.0% | 0.033 | 1 | 0.856 |
| no intervention | 6.1% | 2.0% | 1.394 | 1 | 0.238 |
| Barriers: Reasons not to address drinking (rather or very true):  Shortage of time | 11.5% | 11.6% | 0.001 | 1 | 0.978 |
| I already know many of the women and their habits from previous pregnancies | 24.8% | 8.5% | 5.937 | 1 | 0.015 |
| Most women already know the risks | 36.1% | 29.2% | 0.817 | 1 | 0.366 |
| Women with children are generally well informed about the risks | 36.4% | 27.7% | 1.291 | 1 | 0.256 |
| It is not within my area of responsibility | 4.3% | 2.1% | 0.516 | 1 | 0.473 |
| Uncertainty about clinical relevance of alcohol use | 11.3% | 16.7% | 1.021 | 1 | 0.312 |
| Uncertainty about being able to intervene effectively | 26.2% | 33.3% | 0.988 | 1 | 0.320 |
| Giving advice to drinkers is not effective | 28.9% | 17.8% | 2.299 | 1 | 0.129 |
| Pregnant women probably do not honestly report on drinking | 43.1% | 39.1% | 0.239 | 1 | 0.625 |
| In vocational training I was not informed on the risks of drinking | 17.9% | 4.1% | 5.837 | 1 | 0.016 |
| Drinking in pregnancy is a matter of private life | 2.9% | 0.0% | 1.469 | 1 | 0.226 |
| Screening and counseling cannot be charged | 18.8% | 11.1% | 1.503 | 1 | 0.220 |
| Advice given regarding alcohol consumption:  strict abstinence | 56.6% | 62.7% | 0.649 | 1 | 0.420 |
| never drink more than just sipping | 46.8% | 47.1% | 0.001 | 1 | 0.974 |
| reasonable consumption | 12.8% | 9.8% | 0.342 | 1 | 0.558 |
| no recommendation | 2.6% | 5.9% | 1.524 | 1 | 0.217 |
| Effectiveness of own advice (rather/very high) | 57.3% | 59.5% | 0.072 | 1 | 0.789 |
| Familiar with fetal disorders:  Alcohol embryopathy | 84.3% | 76.5% | 1.789 | 1 | 0.181 |
| Fetal Alcohol Syndrome (FAS) | 81.7% | 92.2% | 3.335 | 1 | 0.068 |
| Fetal Alcohol Effects (FAE) | 26.8% | 27.5% | 0.009 | 1 | 0.925 |
| Alcohol Related Nervous System Disorder | 26.0% | 31.4% | 0.625 | 1 | 0.429 |
| None | 15.3% | 17.6% | 0.171 | 1 | 0.679 |
| 1 glass of alcoholic beer per day during breastfeeding encourages milk production (rather/very true) | 28.8% | 25.5% | 0.200 | 1 | 0.655 |
| 1 glass of alcoholic beer per day during breastfeeding is harmful for the baby (rather/very true) | 82.4% | 84.3% | 0.109 | 1 | 0.741 |
| 1 glass of alcoholic beer per day during breastfeeding may be recommended (rather/very true) | 4.4% | 5.9% | 0.204 | 1 | 0.652 |

| Table S5. Smoking prevention in prenatal care & knowledge of the Guideline of the Swiss Midwives Association by work setting | | | | | |
| --- | --- | --- | --- | --- | --- |
|  | Hospital  (n=89) | Self-employed  (n=193) | Chi^2^ | df | p |
| Variables | Percentage in (%) | |  |  |  |
| Risk perception: 1-2 cigarettes/day  harmless for the child | 5.7% | 7.9% | 3.338 | 2 | 0.188 |
| slightly risky for the child | 55.7% | 64.2% |  |  |  |
| significantly risky for the child | 38.6% | 27.9% |  |  |  |
| Risk perception: 3-9 cigarettes /day |  |  |  |  |  |
| slightly risky for the child | 18.0% | 13.6% | 0.907 | 1 | 0.341 |
| significantly risky for the child | 82.0% | 86.4% |  |  |  |
| Risk perception: 10 or more cigarettes /day |  |  |  |  |  |
| significantly risky for the child | 100.0 | 100.0 | N/C |  |  |
| Risk perception: sudden cessation  not risky for the child | 34.9% | 36.0% | 0.621 | 3 | 0.892 |
| slightly risky for the child | 53.5% | 53.8% |  |  |  |
| significantly risky for the child | 5.8% | 3.8% |  |  |  |
| I don't know | 5.8% | 6.5% |  |  |  |
| Risk perception: Passive smoking  Environmental smoke is a risk | 93.2% | 97.9% | 3.814 | 1 | 0.051 |
| ‘I don’t know’ | 6.8% | 2.1% |  |  |  |
| Screening: Routinely asking all women whether they smoke  all women | 92.1% | 88% | 4.827 | 2 | 0.089 |
| only those suspected for smoking | 4.5% | 11% |  |  |  |
| none | 3.4% | 1.0% |  |  |  |
| Screening: Asking about exposure to passive smoking | 34.1% | 52.4% | 7.853 | 1 | 0.005 |
| Screening: Asking whether the partner smokes | 46.5% | 67.9% | 11.361 | 1 | 0.001 |
| Perceived importance of partner’s smoking (rather or very important) | 92.0% | 92.6% | 0.039 | 1 | 0.843 |
| Routinely explaining the risks of smoking for the child to all women | 44.9% | 56.1% | 3.012 | 1 | 0.083 |
| Stop smoking interventions with smokers  explaining the risks for the child | 88.8% | 83.9% | 1.142 | 1 | 0.285 |
| repeatedly addressing smoking in consequent appointments | 37.1 % | 52.3% | 5.682 | 1 | 0.017 |
| assisting in elaboration of a plan to stop smoking | 25.8% | 42.5% | 7.221 | 1 | 0.007 |
| providing information material to smokers | 28.1% | 20.7% | 1.863 | 1 | 0.172 |
| referral to an expert | 21.3% | 20.7% | 0.014 | 1 | 0.905 |
| referral to behavioral therapy | 2.2% | 6.7% | 2.437 | 1 | 0.119 |
| agreement to quit | 2.2% | 4.1% | 0.641 | 1 | 0.423 |
| nicotine replacement therapy | 9.0% | 6.7% | 0.449 | 1 | 0.503 |
| use e-cigarettes | 5.6% | 4.7% | 0.118 | 1 | 0.732 |
| no intervention | 0.0% | 2.6% | 2.347 | 1 | 0.125 |
| Barriers: Reasons not to address smoking (rather or very true)  shortage of time | 24.4% | 9.5% | 8.961 | 1 | 0.003 |
| I already know many of the women and their smoking habits from previous pregnancies | 18.8% | 40.4% | 11.782 | 1 | 0.001 |
| most women already know the risks | 38.2% | 43.6% | 0.704 | 1 | 0.402 |
| women with children are generally well informed about the risks | 43.2% | 35.5% | 1.470 | 1 | 0.225 |
| It is not within my area of responsibility | 2.3% | 5.2% | 1.151 | 1 | 0.283 |
| uncertainty about clinical relevance of smoking | 12.5% | 15.4% | 0.365 | 1 | 0.546 |
| uncertainty about being able to intervene effectively | 38.1% | 31.4% | 1.136 | 1 | 0.287 |
| giving advice to smokers is not effective | 56.5% | 41.5% | 5.101 | 1 | 0.024 |
| pregnant women probably do not honestly report on smoking | 64.4% | 36.4% | 18.222 | 1 | 0.000 |
| In vocational training I was not informed on the risks of smoking | 18.4% | 23.0% | 0.728 | 1 | 0.394 |
| smoking in pregnancy is a matter of private life and should not be interfered with | 1.1% | 4.0% | 1.645 | 1 | 0.200 |
| screening and counseling cannot be charged | 14.7% | 22.6% | 2.037 | 1 | 0.154 |
| Advice given regarding smoking:  to quit | 77.5% | 76.7% | 0.024 | 1 | 0.876 |
| to reduce | 66.3 % | 70.5% | 0.497 | 1 | 0.481 |
| Use e-cigarettes | 1.1% | 1.6% | 0.081 | 1 | 0.776 |
| not to change | 1.1% | 0.5% | 0.317 | 1 | 0.573 |
| Knowledge of the Guideline of the Swiss Midwives Association & Use of Intervention Strategies^1^ |  |  |  |  |  |
| Familiar with the guideline (yes/no) | 25.9% | 35.4% | 2.379 | 1 | 0.123 |
| Use the intervention strategies recommended in the guideline | 22.7% | 21.8% | 0.022 | 1 | 0.881 |
| Use the 'Stages of Change' model of behavior change | 4.8% | 3.5% | 1.300 | 2 | 0.522 |
| Use the '5A Method' | 2.4% | 3.6% | 3.096 | 2 | 0.213 |
| Use the method of Motivational Interviewing | 55.4% | 52.1% | 0.316 | 2 | 0.854 |
| Frequency of consulting a specialist (one to more than five times a year) | 45.0% | 16.6% | 22.985 | 1 | 0.000 |

| Table S6. Prevention of alcohol consumption in prenatal care by work setting | | | | | |
| --- | --- | --- | --- | --- | --- |
|  | Hospital  (n=89) | Self-employed  (n=193) | Chi^2^ | df | p |
| Variables | Perecentage in (%) | |  |  |  |
| Risk perception: rarely sipping on a glass of alcohol  harmless | 50.0% | 66.5% | 9.543 | 2 | 0.008 |
| slightly risky | 36.9% | 29.1% |  |  |  |
| significantly risky | 13.1% | 4.5% |  |  |  |
| Risk perception: 3 glasses/week  harmless | 2.4% | 1.6% | 2.698 | 2 | 0.259 |
| slightly risky | 24.7% | 34.6% |  |  |  |
| significantly risky | 72.9% | 63.7% |  |  |  |
| Risk perception: 1 glass/day  slightly risky | 3.5% | 6.0% | 0.737 | 1 | 0.391 |
| significantly risky | 96.5% | 94.0% |  |  |  |
| Risk perception: Sporadically drinking large amounts (4 glasses/occasion)  slightly risky | 3.6% | 1.6% | 0.978 | 1 | 0.323 |
| significantly risky | 96.4% | 98.4 |  |  |  |
| Screening: Routinely asking all women whether they consume alcohol  all | 81.2% | 81.3% | 0.125 | 2 | 0.940 |
| only those suspected for drinking | 14.1% | 14.8% |  |  |  |
| none | 4.7% | 3.8% |  |  |  |
| Screening: Specific questions asked regarding alcohol:  Frequency of drinking occasions | 93.8% | 93.1% | 0.042 | 1 | 0.838 |
| Average amount of alcohol consumed | 71.6% | 76.6% | 0.728 | 1 | 0.393 |
| Frequency of binge drinking (4 glasses on a single occasion) | 12.3% | 18.3% | 1.425 | 1 | 0.233 |
| Type of alcoholic beverages consumed | 39.5% | 49.1% | 2.069 | 1 | 0.150 |
| Screening: Asking whether the partner drinks | 14.1% | 33.5% | 10.938 | 1 | 0.001 |
| Perceived importance of partner’s drinking (rather or very important)^1^ | 70.0% | 75.7% | 0.933 | 1 | 0.334 |
| Routinely explaining the risks of alcohol consumption for the child to all women | 55.3% | 66.1% | 2.887 | 1 | 0.089 |
| Stop drinking interventions when a woman drinks:  Explaining the risks for the child | 89.4% | 89.0% | 0.010 | 1 | 0.922 |
| Repeatedly addressing drinking in consequent appointments | 48.2% | 52.2% | 0.364 | 1 | 0.546 |
| Assisting in elaboration of a plan to stop or reduce drinking | 20.0% | 23.6% | 0.437 | 1 | 0.508 |
| Providing information material to drinkers | 23.5% | 24.2% | 0.013 | 1 | 0.908 |
| Referral to an expert | 41.2% | 51.6% | 2.544 | 1 | 0.111 |
| no intervention | 7.1% | 4.9% | 0.488 | 1 | 0.485 |
| Barriers: Reasons not to address drinking (rather or very true):  Shortage of time | 14.9% | 10.7% | 0.781 | 1 | 0.377 |
| I already know many of the women and their habits from previous pregnancies | 11.3% | 27.8% | 8.453 | 1 | 0.004 |
| Most women already know the risks | 34.1% | 34.6% | 0.005 | 1 | 0.945 |
| Women with children are generally well informed about the risks | 32.5% | 35.4% | 0.204 | 1 | 0.652 |
| It is not within my area of responsibility | 3.7% | 3.7% | 0.000 | 1 | 1.000 |
| Uncertainty about clinical relevance of alcohol use | 15.2% | 12.1% | 0.439 | 1 | 0.508 |
| Uncertainty about being able to intervene effectively | 26.8% | 28.0% | 0039 | 1 | 0.844 |
| Giving advice to drinkers is not effective | 32.1% | 24.7% | 1.424 | 1 | 0.233 |
| Pregnant women probably do not honestly report on drinking | 55.0% | 36.6% | 7.357 | 1 | 0.007 |
| In vocational training I was not informed on the risks of drinking | 13.4% | 17.0% | 0.518 | 1 | 0.472 |
| Drinking in pregnancy is a matter of private life | 1.2% | 2.5% | 0.440 | 1 | 0.507 |
| Screening and counseling cannot be charged | 11.0% | 20.1% | 2.927 | 1 | 0.087 |
| Advice given regarding alcohol consumption:  strict abstinence | 63.5% | 54.6% | 1.901 | 1 | 0.168 |
| never drink more than just sipping | 41.2% | 50.3% | 1.932 | 1 | 0.165 |
| reasonable consumption | 7.1% | 14.1% | 2.728 | 1 | 0.099 |
| no recommendation | 3.5% | 2.7% | 0.138 | 1 | 0.710 |
| Familiar with fetal disorders:  Alcohol embryopathy | 81.2% | 82.7% | 0.093 | 1 | 0.761 |
| Fetal Alcohol Syndrome (FAS) | 90.6% | 81.6% | 3.565 | 1 | 0.059 |
| Fetal Alcohol Effects (FAE) | 25.9% | 27.6% | 0.084 | 1 | 0.772 |
| Alcohol Related Nervous System Disorder | 28.2% | 27.0% | 0.043 | 1 | 0.836 |
| None | 21.2% | 12.4% | 3.457 | 1 | 0.063 |
| 1 glass of alcoholic beer per day during breastfeeding encourages milk production (rather/very true) | 25.3% | 30.2% | 0.640 | 1 | 0.424 |
| 1 glass of alcoholic beer per day during breastfeeding is harmful for the baby (rather/very true) | 84.5% | 81.7% | 0.325 | 1 | 0.569 |
| 1 glass of alcoholic beer per day during breastfeeding may be recommended (rather/very true) | 7.1% | 3.9% | 1.271 | 1 | 0.260 |
